# Relationship Between Income Level and Hospitalization Rate in COVID-19 Cases; an Example of Social Factors Affecting Health

**DOI:** 10.22037/aaem.v10i1.1600

**Published:** 2022-04-09

**Authors:** Ali Maher, Hamed Dehnavi, Elham Salehian, Mona Omidi, Khatereh Hannani

**Affiliations:** 1 Department of Health Management and Economics, Virtual School of Medical Education and Management, Shahid Beheshti University of Medical Sciences, Tehran, Iran.; 2 Resources Development Deputy, Shahid Beheshti University of Medical Sciences, Tehran, Iran.

**Keywords:** COVID-19, Iran, Income, Socioeconomic Factors, Health Services Accessibility

## Abstract

**Introduction:**

Considering the population's socioeconomic status and clinical features is essential in planning and performing interventions related to disease control. The main purpose of this study was to investigate the relationship between income level and hospitalization rate of COVID-19 patients‌.

**Methods:**

A cross-sectional study was performed on 198,944 hospitalized COVID-19 patients in Tehran province between March 2020 and March 2021. Data of hospitalized COVID-19 patients was obtained from the Hospital Intelligent Management System (HIM). The income data of patients were obtained from the Iranian Database on Targeted Subsidies belonging to the Ministry of Cooperatives, Labor, and Social Welfare. Data analyses were performed using SPSS software.

**Results:**

About 2.5% of the inpatients were from the first decile, while 20.6% were from the tenth. The share of the lower three deciles of total hospitalization was about 11%, while the share of the upper three deciles was 50%. There was a big difference between the upper- and lower-income deciles regarding death rates. In the first decile, 30% of inpatients died, while the proportion was 10% in the tenth decile. There was a significant and positive relationship between income decline and hospitalization (r = 0.75; p = 0.02). Also, there was a significant and negative relationship between income decline and death rate (r = -0.90; p = 0.01).

**Conclusion:**

Low-income groups use fewer inpatient services, are more prone to severe illness and death from COVID-19‌, and treatment in this group has a lower chance of success. Using a systemic approach to address socioeconomic factors in healthcare planning is crucial.

## 1. Introduction:

 On December 8, 2019, China reported a type of coronavirus that caused an infectious disease with symptoms such as fever, cough, and shortness of breath. On January 6, 2020, the World Health Organization named the disease COVID-19 (1). More than 220 million confirmed cases have been reported since the epidemic began, and more than 4.5 million people have died. The highest rates of death have been reported in the United States, Europe, and Southeast Asia (2). The effects of this crisis in various dimensions are not yet fully known. The World Health Organization has called COVID-19 a humanitarian crisis and described it as more than a health crisis. Estimates have shown that COVID-19 is likely to increase global poverty and inequality, so achieving sustainable development goals (SDGs) will certainly require more effort (3). 

COVID-19 has cost the world more than $10 trillion so far, and more than 10 million people worldwide are affected by poverty for every percentage drop in the global economy (4). Nearly 80% of the world's population lives in low- and middle-income countries (LMICs) and, therefore, faces extra pressure because there is not sufficient infrastructure and facilities (5). Consequently, it is crucial to find effective strategies in each country.

Health is related to the socioeconomic status (SES) of demographic groups in complex ways. In other words, inequality in social determinants of health (SDH) leads to inequality in health. Inequality in health exists not only between the rich and the poor, but at every level of socioeconomic status (6, 7).

 A review of data from 178 countries/regions indicates that socioeconomic factors can significantly affect the risk of COVID-19 (8). Poor people who do not usually have access to health services are more vulnerable in times of crisis (4). Low socioeconomic status (SES) is widely associated with disease and mortality. People with lower SES are more likely to be affected by COVID-19 (9-13). Studies in various countries, including the United States (5), Brazil (14), Africa (15), the United Kingdom (16), Sweden (10), Scotland (17), and France (18), confirm this claim. Based on these studies, sex (9, 10), education (10), income (5, 10, 19), place of residence (city or village) (9), race and ethnicity (11, 12, 20), underlying diseases (21) and living in areas with high population density (22) affect the incidence and mortality of COVID-19. In general, when the level of SES improves, the severity and complications of the disease decrease (23). Thus, the interaction of COVID-19 and socioeconomic status creates a combination that imposes the burden of inequality on disadvantaged people in society (10).

The first cases of coronavirus in Iran were reported in late February 2020. By July 2021, five waves of disease outbreaks have occurred. To date, about 5 million cases have been confirmed, and approximately 110,000 patients have died from the disease. Iran has faced many challenges in early dealing with the disease. Political and economic sanctions have greatly affected the country's economy. In Iran, infrastructure development, capacity building, and resource allocation to control the epidemic and treatment of patients face many challenges (24). Concerns about protecting vulnerable groups have increased in the wake of the COVID-19 crisis, followed by the global economic downturn and countries' focus on ensuring population health (22). Considering the relationship between socioeconomic factors of patients and COVID-19 mortality is crucial to targeting vulnerable groups (25). A study conducted in Tehran demonstrated that those with lower economic status were more at risk of morbidity and mortality (26). Despite the studies conducted in Iran on the effect of socioeconomic factors, there is no study that clearly shows the impact of income yet. We aimed to investigate relationship between COVID-19 mortality and income level of patients. The findings of this study can highlight the gap in access to and utilization of health care services due to socioeconomic differences in Tehran province. 

## 2. Methods:

**2.1. Study design and setting**

This cross-sectional study was performed on hospitalized COVID-19 patients in Tehran province. Tehran province has an area of about 13,000 square kilometers, and according to the official census, in 2016, its population was 13,267,000. About 16.6% of the country's population lives in Tehran province. Health services in Tehran province are provided by three medical universities: Shahid Beheshti, Tehran, and Iran. About 17% of hospitals and 22% of hospital beds in the country are located in Tehran province (27). 

The protocol of the study was approved by Ethics committee of Shahid Beheshti University of Medical Sciences (Ethics code: IR.SBMU.SME.REC.1400.086) and the researchers adhered to the confidentiality of patients’ profiles.

**2.2.** **Data collection and procedure**

The study sample consists of all inpatients with positive PCR hospitalized from March 20, 2020, to March 20, 2021 in Tehran province. We obtained inpatient information from the hospital intelligent management system (HIM) of Shahid Beheshti University of Medical Sciences. The intelligent hospital management system is a management information system (MIS) that aggregates data from various systems and provides them in dashboards and reports for managers. The data of COVID-19 patients were collected directly from the hospital information system (HIS). All the data recorded in the hospital were directly entered into the study and there was no missing data in the data collection stage. The income data of patients were obtained from the Iranian Ministry of Cooperatives, Labor, and Social Welfare. Patients' income and hospitalization information were matched using a national code. The Statistics Center of Iran categorizes families into ten groups based on total annual income in the annual household budget report. The first decile is the lowest income group, and the tenth decile is the highest income group of families. The use of HIM data was done after receiving ethical approval from Shahid Beheshti University of Medical Sciences regarding the use of data.

**2.3. Definition**

In its annual household budget report, the Statistics Center categorizes households into ten groups based on total annual income and expenses. The first decile is the lowest income group, and the tenth decile is the highest income group of families. The Statistics Center examines each decile's types of costs and characteristics and announces them in different tables. In 2021, if the total cost of a household is about $64 per month, this family will be in the first decile and is among the poorest 10% of society. The cost of a household is about $640 in the tenth decile, which consists of the richest 10% of people in society (28).

**2.4. Data analysis**

The data were assessed in several steps. The frequencies of patients were calculated in terms of income deciles. SPSS software version 24 was used to perform calculations and data analysis. Descriptive statistics and frequency distribution were used as appropriate. Spearman's correlation coefficient was used to test the significance of the hospitalization and death rate differences between income deciles. Results were presented in graphs and tables. 

## 3. Results:

Over the study period, 198,944 patients were hospitalized; 88% (n=175,099) recovered and were discharged from the hospital, and 12% (n=23845) died (Table 1). With increase in income, the number of patients also increased. The number of hospitalizations in the first income decile was 4,920 (2.5% of total hospitalization), and in the tenth decile was 40,954 (20.6% of total hospitalization). The number of hospitalizations in the tenth decile was 8.3 times that of the first decile. In the same way, the number of death and recovered patients also increased with rise in the income decile. The share of the lower three deciles was 11.1% of the total hospitalizations, while the share of the upper three deciles was 50%. 

**Figure 1:**
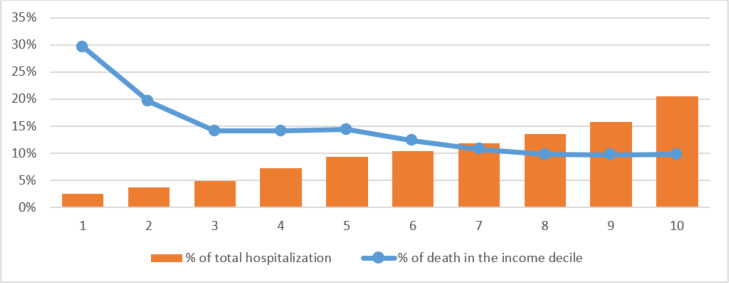
Income deciles and rate of hospitalization and mortality among COVID-19 cases

The death rates decreased from the deprived decile to the rich decile. There was a big difference between the deprived and wealthy deciles in terms of death rates. In the first decile, 30% of hospitalized patients died, while the rate was 10% in the tenth decile. The death rate in the studied province was 12%. Death rates in the upper seven deciles were between 10 and 14%. As Figure 1 shows, there were two opposing trends between the death in income deciles and their share of total hospitalization. The first decile has the lowest hospitalization rate (2.5%), while the highest death rate belonged to this decile (30%). The tenth decile has the highest hospitalization rate (20.6%), while it has the lowest death rate (9.8%). There was a significant and positive relationship between income decline and share of total hospitalization (r = 0.75; p = 0.02). Also, there was a significant and negative relationship between income decline and death rate (r = -0.90; p = 0.01). 

## 4. Discussion:

This study aimed to investigate the relationship between income deciles and the number of hospitalizations and deaths among COVID-19 cases. The results showed that inpatient service utilization had increased with the increase in income. On the other hand, more low-income people died because they could not afford it. Health is considered one of the fundamental human rights. A comprehensive approach to health care is closely related to social and economic conditions, physical environment, and lifestyle. The Commission on Social Factors Affecting Health considers health inequalities to be the result of decades of gradual exposure to health risks among those who have lived in deprivation (19). Several studies have reported a higher prevalence of non-communicable diseases (NCDs) in disadvantaged groups compared to populations with higher socioeconomic status (15, 29). Inequalities were rising in many parts of the world before the COVID-19 epidemic. The current crisis seems to be increasing these inequalities (30). Vulnerability is one of the main components of getting exposed to the risk and influence of COVID-19. Groups in the community are not equally affected by diseases. Vulnerable groups, including the elderly and those with underlying diseases, have higher mortality rates. COVID-19 highlights the societal gaps and is likely to deepen existing inequalities (16). Socioeconomic status (SES) can expose people to disease and death in various ways. People's jobs can expose them to more viruses and infections. In addition, smoking and a poor diet that reduces the body's immune system will increase the risk of infection (13). A study by Su et al. using data from 178 countries/regions showed that socioeconomic factors could be significantly associated with the risk of COVID-19. A study in Tehran, which examined the Spatio-temporal patterns of the epidemic, showed that demographic composition and major physical characteristics at the neighborhood level were important factors explaining the high rate of infection and mortality. Population density and facility distribution patterns were the most critical factors in this regard. Hot spots were observed in areas with low socioeconomic status (26). Another study in Tehran showed that health awareness level was higher in areas with high socioeconomic status (northern part of the city), and the higher death rate in the eastern parts of Tehran could be associated with lower socioeconomic status; since people with low socioeconomic status seek health services only in the advanced stages of the disease (31). A study conducted by Clouston et al. in the United States showed that cities with a high percentage of high-risk residents, including minors and the elderly had higher mortality rates. Residents of cities with higher SES were at lower risk of COVID-19 mortality than those living in cities with low SES (32). Additionally, in Chicago about 70 percent of deaths occurred in black people, while they made up only 30 percent of the population. (30). This study showed that the death rate of patients in low-income deciles in the province is higher. A large number of studies reported similar results. A survey of the Health Transformation Plan (HTP) impact in Iran on catastrophic costs showed that the provinces had different Catastrophic health expenditure (CHE) rates, which may be affected by various socioeconomic factors. Literacy and employment rates should be increased to protect patients from catastrophic health expenditure (32, 33). 

A study by Sesé et al. in Seine-Saint-Denis (SSD), France, the poorest area of Paris, showed that the cause of higher death rates in this area was lower SES (18). A study by Navaratnam et al. on COVID-19 inpatients in UK hospitals showed that more deprivation was associated with a higher risk of death (34). Various authors, including Wilkinson, show that people's health is a function of their income (absolute income hypothesis). Regarding income inequality, the relative income hypothesis shows that income inequality has a negative impact on population health. Inequality is recognized as a critical risk factor for community health (19, 35). The evidence clearly shows that there is a non-linear relationship between health and income. Accordingly, redistributing income from the rich to the disadvantaged can help improve health indicators (19). 

Low income affects life in many ways. Living in more deprived neighborhoods and housing conditions (especially small or overcrowded housing) increases the risk of infection (13). A study in New York wards found that the rate of hospitalization and mortality was lowest among residents of Manhattan's most affluent area, where the population is predominantly white (20). In Sweden, a study by Rafal et al. showed that being a man, having a lower personal income, lower education, and being single increased the risk of death from COVID-19 (10). A study by Rose et al. in the United Kingdom on the relationship between income levels and deaths from COVID-19 showed that each percent increase in the proportion of the population experiencing income deprivation was associated with a 2% increase in deaths from COVID-19 (30).

A study by Lone et al. in Scotland on COVID-19 patients admitted to the intensive care unit (ICU) showed that patients living in disadvantaged socioeconomic areas were more likely to need ICU admission and had higher mortality rate after 30 days. ICU wards in more deprived areas had higher bed occupancy rate (BOR), and the average length of stay (ALOS) was higher (17). People who have limited access to health care and have COVID-19 symptoms may delay their treatment or even hide their sickness. As a result, they may seek health services only in the late stage of the disease. This condition reduces the chance of recovery and increases the likelihood of transmission to others (22). Although it has been argued that all countries should pursue a quarantine policy to prevent the rapid spread of the disease due to the irreversible consequences of the pandemic, low-income people are forced to go out of home due to economic pressures (36). Those who live in areas with higher socioeconomic deprivation are more likely to live in crowded neighborhoods that are more prone to disease. More people have non-communicable diseases (NCD), such as high blood pressure, obesity, diabetes, and heart disease in socioeconomically disadvantaged areas. These factors increase the severity of the disease and raise the need for ICU admission and the risk of death (17). 

This study demonstrated that low-income groups use fewer inpatient services, but more hospitalized COVID-19 patients die from the disease. One reason is the treatment-seeking behavior of this group. Minorities and disadvantaged people pay less attention to the disease and delay treatment (37). Disease control and early detection are essential in controlling COVID-19 before the disease progresses; otherwise, patients are less likely to survive. 

**Table 1:** Frequency of hospitalization and mortality of COVID-19 patients based on the income decile

**Deceased**	**Recovered**	**Hospitalized**	**Income decile**
1460 (6.1)	3460 (2.0)	4920 (2.5)	**1 **
1454 (6.1)	5997 (3.4)	7451 (3.7)	**2**
1374 (5.8)	8349 (4.8)	9723 (4.9)	**3**
2040 (8.6)	12339 (7.0)	14379 (7.2)	**4**
2702 (11.3)	16019 (9.1)	18721 (9.4)	**5**
2550 (10.7)	18196 (10.4)	20746 (10.4)	**6**
2543 (10.7)	21063 (12.0)	23606 (11.9)	**7**
2639 (11.1)	24289 (13.9)	26928 (13.5)	**8**
3058 (12.8)	28458 (16.3)	31516 (15.8)	**9**
4025 (16.9)	36929 (21.1)	40954 (20.6)	**10**
23845 (100)	175099 (100)	198944 (100)	**Total**

Data are presented as number (%).

## 5. Limitations

This study has a limitation. Data related to patients' income deciles were not provided directly to the research team due to confidentiality. It was impossible to analyze the patients' income data one by one. Data matching was performed using the national code. For this purpose, the national code of patients was sent to the Iran Ministry of Cooperatives, Labor, and Social Welfare. The experts of that organization matched the data with confidentiality.

## 6. Conclusion

The findings of this study demonstrated that low-income groups use fewer inpatient services, and treatment in this group has a lower chance of success. Planning to identify vulnerable people in the early stages of the disease and increase their access to health services will help improve community health and reduce health sector costs in Tehran.

## 7. Recommendations for future research

Further studies on why low-income groups do not use inpatient services and the reasons for delays in receiving services can complement the findings of this study and help interpret the findings. 

## 8. Clinical implications for health managers and policymakers

Paying attention to low-income deciles in Tehran province and identifying their health needs should be one of the priorities of policymakers and managers of the health system. Targeting available resources and building more capacity based on socioeconomic studies is vital in managing and ending the COVID-19 epidemic.

## 9. Abbreviations

SDGs: Sustainable Development Goals

SES: Socioeconomic Status

SDH: Social Determinants of Health

NCDs: Non-Communicable Diseases

HTP: Health Transformation Plan

BOR: Bed Occupancy Rate

ALOS: Average Length of Stay

## 10. Declarations:

### 10.1. Acknowledgements

The authors thank the reviewers who helped improve the article with their comments. The ethics committee approval code is IR.SBMU.SME.REC.1400.086.

### 10.2. Authors' contributions

AH designed the study. AH, KH and ES collected the data. AH, HD analyzed and interpreted the data. AH, HD and MO drafted the manuscript. All authors have read and approved the final manuscript. 

### 10.3. Availability of data and materials

The data that support the findings of this study are available from the Iranian Ministry of Health. However, restrictions apply to the availability of these data, which were used under license for the current research, and so are not publicly available. However, data are available from the authors upon reasonable request and with permission of Iranian Ministry of Health.

### 10.4. Competing interests

The authors declare that they have no competing interests

### 10.5. Funding

No funds were used for the study.
